# Southern Surgical and Gynecological Association

**Published:** 1896-11

**Authors:** 


### Society Notes.

### Southern Surgical and Gynecological Association.

The ninth annual meeting of the Association will be held in Nashville, Tenn., Tuesday, Wednesday and Thursday, November 10th, 11th and 12th, 1896. The Nicholson House has been selected as headquarters for the Association. The rates will range from $2.00 to $4.00 per day. Arrangements have been made with the Southern Passenger Association for reduced rates on the certificate plan. These certificates, properly signed, will entitle those who atteud to a return ticket at the rate of one-third of one full fare. Those who contemplate attending the Pan-American Medical Congress, to be held in the City of Mexico, November 16–19, will have time to do so after the meeting of the Southern Surgical and Gynecological Association. A rate of one fare for the round trip has been made on account of the Congress, stopping over privileges being allowed holders of tickets therefor.

The following is a partial list of the papers to be read:

1. President's Address—E. S. Lewis, M. D., New Orleans, Louisiana.

2. Memorial Address on Dr. Paul F. Eve—Richard Douglas, M. D., Nashville, Tenn.

3. Acute mania following surgical operations—Joseph Price, M. D., Philadelphia, Pa.

4. Cholelithiasis, with report of cases—A. M. Cartledge, M. D., Louisville, Ky.

5. Treatment of carcinoma uteri—Jas. T. Jelks, M. D., Hot Springs, Ark.

6. Maternal impressions and their influence upon the fœtus in utero, illustrated by photographs—C. H. Mastin, M. D., Mobile, Ala.

7. Ureteral anastomosis—J. W. Bovee, M. D., Washington, D. C.

8. Compound comminuted fracture of the radius and ulnar, near the wrist joint—H. M. Hunter, M. D., Union Springs, Ala.

9. Enchondroma of the pelvis, with report of a successful operation—Rudolph Matas, M. D., New Orleans, La.

10. The treatment of pregnancy and labor complicated by fibroid tumors of the uterus—Henry D. Fry, M. D., Washington, D. C.

11. Uterine drainage as a factor in the prevention and relief of pelvic inflammation—R. R. Kime, M. D., Atlanta, Ga.

12. Splitting the capsule for nephralgia, with report of cases—George Ben Johnston, M. D., Richmond, Va.

13. Gun shot wounds of the abdomen—W. E. Parker, M. D., New Orleans, La.

14. Vaginal drainage in surgery of the uterus and other pelvic structures—W. H. Wathen, M. D., Louisville, Ky.

15. Mental complications following surgical operations—J. T. Wilson, M. D., Sherman, Texas.

16. Tracheotomy versus intubation—Chalmers Deaderick, M. D., Knoxville, Tenn.

17. The treatment of inevitable abortion—Virgil O. Hardon, M. D., Atlanta, Ga.

18. Some remarks on fractures—W. F. Westmoreland, M. D., Atlanta, Ga.

19. Hypodermoclysis in severe railway injuries requiring multiple amputations—James Evans, M. D., Florence, S. C.

20. Wounds of the liver, with report of cases—S. M. Fortier, M. D., New Orleans, La.

21. Peculiarities of the surgical diseases and injuries of the neck—Edmond Souchon, M. D., New Orleans, La.

22. Clamps instead of ligatures in vaginal hysterectomy—H. S. Cocram, M. D., New Orleans, La.

23. Contributions to the pathology of adherent placenta—Bedford Brown, M. D., Alexandria, Va.

24. The dry method in intra-uterine surgery—Edwin Walker, M. D., Evansville, Ind.

25. Subject to be announced—J. B. S. Davis, M. D., Birmingham, Ala.

26. Subject to be announced—L. S. McMurtry, M. D., Louisville, Ky.

27. Subject to be announced—W. B. Rogers, M. D., Memphis, Tenn.

28. Subject to be announced—Geo. H. Noble, Atlanta, Ga.

29. Subject to be announced—N. P. Dandridge, M. D., Cincinnati, O.

30. Subject to be announced—George J. Engelmann, St. Louis, Mo.

